# Putative type 1 thymidylate synthase and dihydrofolate reductase as signature genes of a novel bastille-like group of phages in the subfamily *Spounavirinae*

**DOI:** 10.1186/s12864-015-1757-0

**Published:** 2015-08-07

**Authors:** Paul Tetteh Asare, Tae-Yong Jeong, Sangryeol Ryu, Jochen Klumpp, Martin J. Loessner, Bryan D. Merrill, Kwang-Pyo Kim

**Affiliations:** Department of Food Science and Technology, College of Agriculture and Life Sciences, Chonbuk National University, Jeonju, Jeollabuk-do 561-756 Korea; Department of Food and Animal Biotechnology, Seoul National University, Seoul, Korea; Department of Agricultural Biotechnology, Center for Agricultural Biomaterials, Seoul National University, Seoul, Korea; Research Institute for Agriculture and Life Sciences, Seoul National University, Seoul, Korea; Institute of Food, Nutrition and Health, ETH Zurich, Schmelzbergstrasse 7, 8092 Zurich, Switzerland; Department of Microbiology and Molecular Biology, Brigham Young University, Provo, UT USA

**Keywords:** *Spounavirinae*, Thymidylate synthase, Dihydrofolate reductase, Bastille-like group, Bacteriophages

## Abstract

**Background:**

*Spounavirinae* viruses have received an increasing interest as tools for the control of harmful bacteria due to their relatively broad host range and strictly virulent phenotype.

**Results:**

In this study, we collected and analyzed the complete genome sequences of 61 published phages, either ICTV-classified or candidate members of the *Spounavirinae* subfamily of the *Myoviridae.* A set of comparative analyses identified a distinct, recently proposed Bastille-like phage group within the *Spounavirinae*. More importantly, type 1 thymidylate synthase (TS1) and dihydrofolate reductase (DHFR) genes were shown to be unique for the members of the proposed Bastille-like phage group, and are suitable as molecular markers. We also show that the members of this group encode beta-lactamase and/or sporulation-related SpoIIIE homologs, possibly questioning their suitability as biocontrol agents.

**Conclusions:**

We confirm the creation of a new genus—the “Bastille-like group”—in *Spounavirinae,* and propose that the presence of TS1- and DHFR-encoding genes could serve as signatures for the new Bastille-like group. In addition, the presence of metallo-beta-lactamase and/or SpoIIIE homologs in all members of Bastille-like group phages makes questionable their suitability for use in biocontrol.

**Electronic supplementary material:**

The online version of this article (doi:10.1186/s12864-015-1757-0) contains supplementary material, which is available to authorized users.

## Background

*Spounavirinae* is a subfamily of the *Myoviridae*, and its members possess a large isometric head (75–100 nm) with a long contractile tail (140–220 nm) [19]. An increasing interest in the *Spounavirinae* members can be noted, due to their broad host range and strictly virulent lifestyle [25]. According to the current ICTV (International Committee on Taxonomy of Viruses) classification, the *Spounavirinae* subfamily comprises two genera (the Spouna [SPO1]-like viruses with modified DNA and shorter tails, and the Twort-like viruses with larger tails and unmodified DNA) and a group of orphan phages (unassigned-group) [18, 19].

Bacteria of the genus *Bacillus* are ubiquitous in nature. The genus includes one of the best characterized model organisms, *B. subtilis,* as well as medically significant human pathogens *B. cereus* (which causes food poisoning) and *B. anthracis* (the causative agent of anthrax) [13, 29]. Phages have been isolated for all members of this genus, providing a unique opportunity to investigate the diversity of phages that infect different hosts within a bacterial genus [13]. As of the date of manuscript submission, 34 large genome *Bacillus Spounavirinae* (*Myoviridae* with genome above 127 kb) have been sequenced and deposited in the NCBI GenBank database, of which only phage SPO1 has been assigned a genus under the current recognized ICTV classification [18]. The remaining phages are considered orphan phages and their taxonomic position is subject of discussion [3, 10, 20]. Recently, a “Bastille-like group” within the *Spounavirinae* clade was proposed, containing eight *Bacillus* phages [3].

Undoubtedly, more *Spounavirinae* phages (or more specifically, Bastille-like phages) will be isolated and there is a need to establish a more defined taxonomic system in order to explore the evolutionary relationships and genetic linkages in these types of phages. The first taxonomic overhaul of the group of phages previously named “SPO1-like phages” occurred some years ago and resulted in the creation of the *Spounavirinae* subfamily with two other groups of phages [19]. The availability of many new phage genome sequences will enable a more concise classification, as well as the identification of many genetic markers.

In this study we collected and analyzed the complete genome sequences of 61 published phages either ICTV-classified or candidate members of the *Spounavirinae* subfamily. We confirm the presence of a distinct cluster (Bastille–like group, now with 26 *Bacillus* phage members) in the subfamily, which prompts for a re-assessment of the taxonomic situation. More importantly, we report Bastille-like group-specific sequences that could serve as a “signature” for identification of members of the proposed group.

## Results

### Comparative genomics identifies new members of Bastille-, SPO1- and Twort-like viruses in the *Spounavirinae* subfamily

#### CLANS analysis

**Fig. 1:**
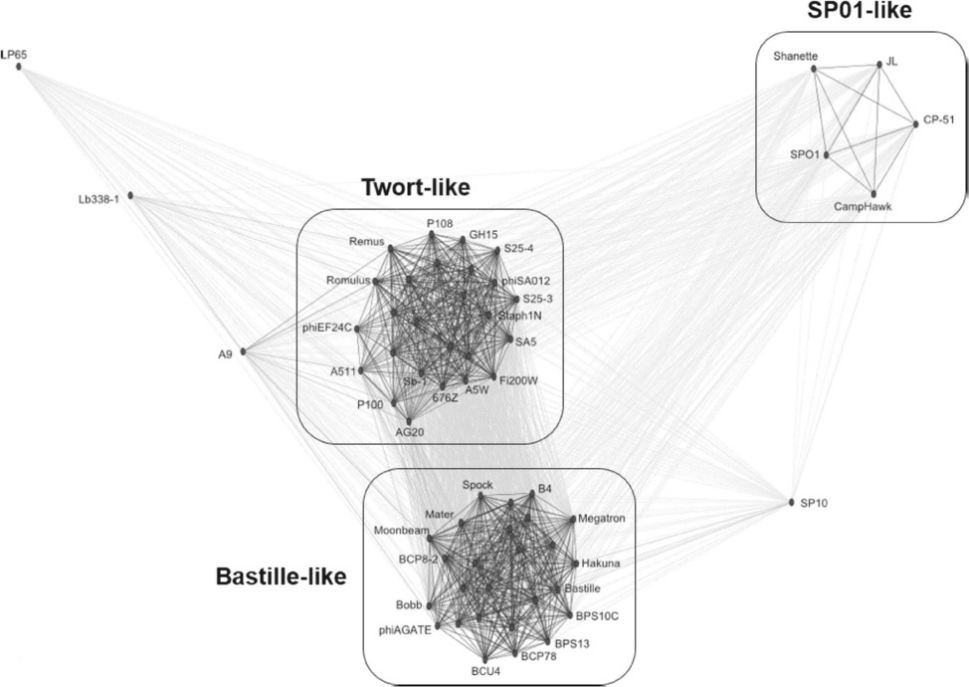
CLANS analysis of a total of 61 phages, eight ICTV classified *Spounavirinae* phages (SPO1, Twort, A511, P100, G1, K, phiEC24C, and LP65), and 53 unclassified *Spounavirinae* candidates (Myoviruses with genome size of >127 kb in NCBI database). Edge weights were calculated from the *P* values of the BLASTn high-scoring segment pairs (e-value cut-off =1e-5). The network was visualized after 25000 runs using the CLANS software package [32]. All analyzed sequences are listed in methodology.

When the genomes of 61 phages (8 ICTV-classified *Spounavirinae* phages and 53 unclassified *Spounavirinae*) were compared using CLANS, three distinct groups were observed (Fig. 1). The first group consists of 26 phages including eight recently-proposed Bastille-like group phages (Bastille, B4, B5S, BCP78, BCU4, BPS13, W.Ph. and phiAGATE) [3]. The second group (26 members) includes the entire ICTV-recognized Twort-like viruses (A511, G1, P100, Twort, and K) in addition to other un-classified phages (GH15, JD007, AG20, phiEF24C, Remus, Romulus and others). The SPO1-like group represents the third group (SPO1, CampHawk, Shanette, CP-51 and JL) and is more distantly related to the other two groups. Phages A9, LP65, Lb388-1 and SP10 were considered as singletons (Fig. 1).

#### Dot plot analysis

**Fig. 2:**
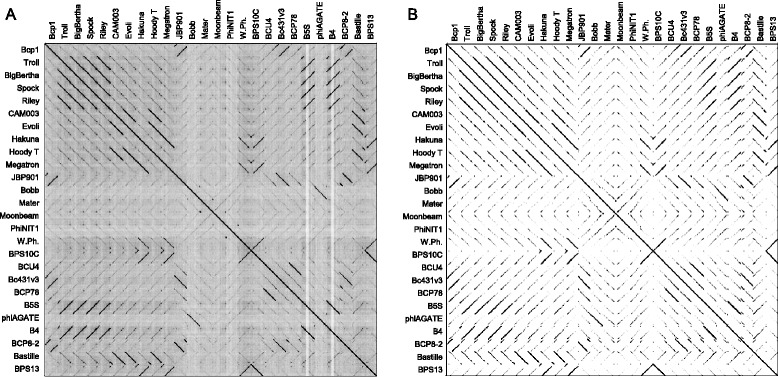
Nucleotide (**a**) and amino acid sequence (**b**) dot plot analysis of 26 Bastille-like group phages in *Spounavirinae*. Dot plots were generated using Gepard [9] and whole amino acid sequence of phages were retrieved from Phamerator [33]

Whole genome nucleotide (Additional file 1: Figure S1A) and amino acid sequence (Additional file 1: Figure S1B) dot plot analysis of the 61 phages also revealed 3 clusters and singletons similar to the CLANS analysis result. More detailed analysis showed that similarity at amino acid level was clearly more obvious than at nucleotide level among Bastille-like group phages (Fig. 2). Similar results were reported previously [13].

#### Phylogenetic study using single gene products

**Fig. 3:**
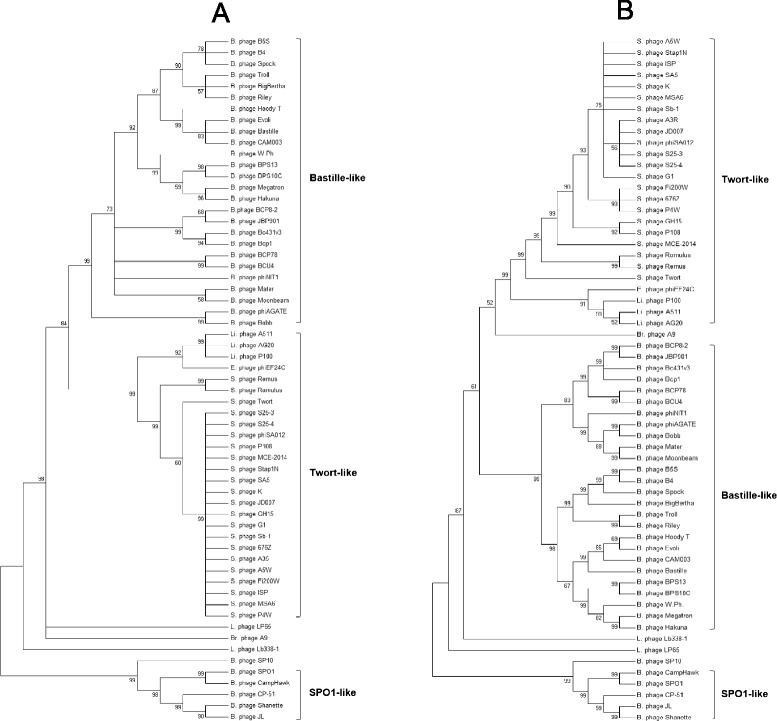
Comparative phylogenetic analysis of major capsid proteins (**a**) and tail sheath proteins (**b**) of 61 *Spounavirinae* phages using MEGA v6 [27] and Muscle programs and the Maximum Likelihood (ML) method. Bootstrapping was set to 1000 and the unrooted tree was collapsed at a less than 50 % bootstrap value. B, *Bacillus*; Br, *Brochothrix*; E, *Enterococcus*; L, *Lactobacillus*; Li, *Listeria*; S, *Staphylococcus*

In order to construct a phylogenetic tree using single gene products, putative major capsid proteins and tail sheath proteins were identified from all 61 phages. When the Maximum Likelihood algorithm was used, three clusters identical to CLANS analysis were observed (Fig. 3a and b).

Large terminase subunit and DNA polymerase sequences were also used for phylogenetic analysis [10, 24, 28]. Interestingly, two genes were not found in the *Staphylococcus* phages Remus and Romulus and it was previously reported that they were fragmented by mobile elements [34]. Thus, when the phylogenetic tree was drawn for the proteins in the remaining 59 phages, the clusters did not correspond to the observed CLANS cluster pattern (data not shown).

### Phamerator analysis identified candidate signature genes that are specific for Bastille-like group phages

A Phamerator database was created using the 61 large genome *Spounavirinae* phages with minimum, maximum and mean genome length of 127065 bp (A9), 165238 bp (BigBertha) and 147716 bp, respectively (Additional file 2). When the chosen parameters were applied to the dataset, the 61 *Spounavirinae* phage genomes containing a total of 13996 gene products were assembled into 3200 phamilies (phams) of which 1464 phams are orphams (45.75 %), or phams with only one gene product. The largest pham (pham 1971) contained 96 members. The mean pham size was 4.37 gene products.

**Fig. 4:**
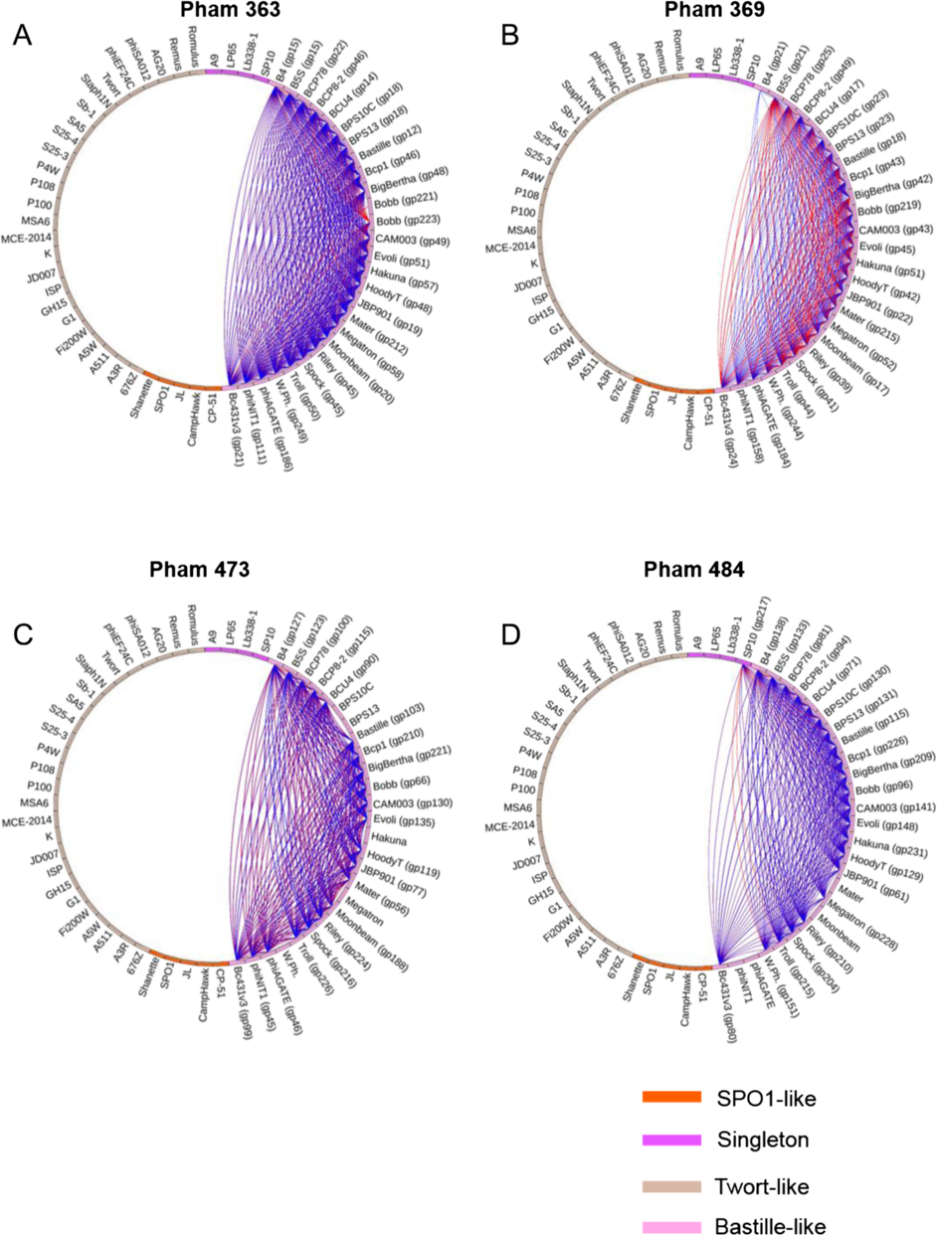
Phamily circles connecting 27 type 1 thymidylate synthase (TS1) gene products in 26 phages (pham 363, **a**), 26 dihydrofolate reductase (DHFR) gene products in 26 phages (pham 369, **b**), 21 DNA segregation ATPase FtsK/SpoIIIE gene products in 21 phages (pham 473, **c**) and), 23 metallo-beta-lactamase gene products in 23 phages (pham 484, **d**). Phamilies were created when gene products exhibited E-values smaller than 1 × 10^−50^ or greater than 32.5 % identity with at least one other gene product in the pham. Phage Bobb encodes two thymidylate synthase homologs (gp221 and gp223) that belong to pham 363 (See Fig. 5 and the text for more details)

In order to select gene products that were conserved in the Bastille-like group phages, phams were identified that only include gene products from Bastille-like phages. These included pham 363 (found in 27 members [Phage Bobb has two; see below]; containing thymidylate synthase domain), pham 365 (26; deoxynucleoside monophosphate kinase domain), pham 369 (26; dihydrofolate reductase [DHFR] domain), pham 473 (21; DNA segregation ATPase FtsK/SpoIIIE domain), pham 484 (23; metallo-beta-lactamase domain), pham 518 (26; holin domain), pham 558 (25; CRISPR/Cas system-associated transcriptional regulator CasRa domain) (Fig. 4 and Additional file 3).

Among those Bastille-like group specific phams, pham 369 was chosen for further studies as no other group of phages in the database contain a DHFR gene in their genome. In addition, pham 363 was also chosen for further studies due to high similarities among the members in the pham. Pham members shared more than 43 % identity and E-values lower than 2.80 × 10^−88^ (See below for more details).

We also analyzed the other Bastille-like specific phams (pham 365, pham 518 and pham 558). However, we concluded that these three phams are not suitable as the signature gene for the group. It is because members of pham 365 exhibit relatively low similarities (minimum amino acid sequence identity 24.2 %; E-value 3.13 × 10^−18^) among the members and have differently annotated gene names (dephospho-CoA kinase, dNMP kinase and adenylate kinase). In the case of pham 518 (a common phage gene product, holin) and pham 558 (transcriptional regulator), homologous proteins are found in non-Bastille group phages and phylogenetic analysis failed to observe a unique cluster for the Bastille-like group (data not shown).

Pham 473 (minimum of 41.4 % identity and an E-value of 0) and pham 484 (minimum of 28.8 % and an E-value of 3.7 × 10^−48^) were characterized further due to their significance in phages as agents in biocontrol (See below) (Fig. 4c and d).

### Four types of thymidylate synthase homologs (TS) are found in *Spounavirinae*, and a TS1 (Type 1 thymidylate synthase homolog) can be used as a signature gene for the Bastille-like group phages

Pham 363 contains 27 gene products from 26 different phages. Phage Bobb has two genes in the pham; see below. These genes encode a Type 1 thymidylate synthase (TS1) (Fig. 4) and are only found in the 26 Bastille-like group phages. Percent identities and E-values among the 26 gene products are 43.2 to100 % and 2.80 x 10^−88^ to 0.0 respectively. When the 26 gene products in pham 363 were BLAST-searched, high percent identity hits (50–53 %) from TS gene products in *Bacillus* spp. (*B. cereus, B. thuringiensis* and *B. mycoides*) were identified.

**Fig. 5:**
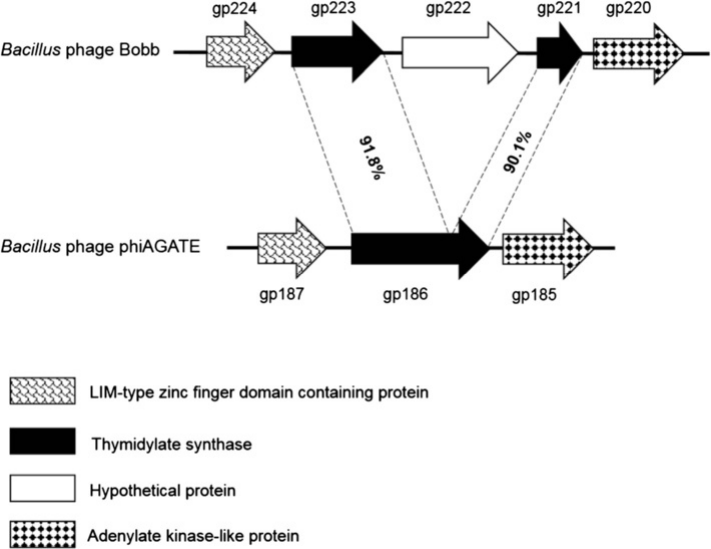
Gene arrangements of the TS and DHFR region with their adjacent genes in Bobb and phiAGATE (not to scale). The numbers given in the connecting lines are the ClustalW alignment values

*Bacillus* phage Bobb contains two members of pham 363, gp221 and 223 (Figs. 4a and 5). This was unusual, since all the other members of the Bastille-like group encode a single thymidylate synthase homolog. Interestingly, protein sequence analysis showed that gp223 (207 a.a. long) and gp221 (101 a.a. long) exhibit 90.1 and 91.8 % identities with N- and C-terminus of phage phiAGATE TS1 (305 a.a long), respectively (Fig. 5). In addition, gp222 present in between gp223 and gp221 in phage Bobb encodes intron endonuclease homolog that contains the N-terminal catalytic domain for GIY-YIG intron endonuclease I-TevI, I-BmoI, I-BanI, I-BthII proteins and a C-terminal YIG family of class I homing endonucleases C-terminus (GIY-YIG_Cterm) (see Discussion for more details). These data suggest that gp221 and gp223 were originally introduced as one complete TS1.

Pham 153 contains one member (Type 2 thymidylate synthase, TS2), gp166 of *Brochothrix* phage A9 (Additional file 4). Gp166 of A9 exhibits 36 % amino acid identity with thymidylate synthase of *Aneurinibacillus aneurinilyticus.* On the other hand, gp166 showed a maximum amino acid sequence identity of 35 % (E-value 1.0 × 10-^46^) with *Bacillus* TS.

Pham 2633 has only one gene member (gp203; Type 3 thymidylate synthase, TS3) and is found in *Enterococcus faecalis* phage phiEF24C, a member of Twort-like group phages. Gp203 in phiEF24C exhibits 36–48 % amino acid identities with TS proteins from *Enterococcus* spp. (*E. raffinosus*, *E. avium* and *E. malodoratus*) and *Aneurinibacillus aneurinilyticus*. On the contrary, gp203 showed 31–43 % (93 % query coverage) identity with *Bacillus* TS proteins.

Pham 1677 has six genes (Type 4 thymidylate synthase, TS4) that are found in all five members of SPO1-like phages (SPO1 gp141, CampHawk gp138, CP-51 gp086, JL gp159 and Shanette gp160) as well as in SP10 (gp188), a member of Twort-like group. They contain a TS domain and are annotated as TS (CampHawk and JL), deoxyuridylate hydroxymethyltransferase (SPO1 and SP10), putative replication protein (CP-51) or hypothetical protein (Shanette) in the NCBI database. The members of this pham share 46.4-100 % identity and E-values from 1.4 × 10^−116^ to 0.0. Phage TS4 proteins are less similar to *Bacillus* TS (33 % identity with 51 % query coverage), and maximum similarities were found with deoxyuridylate hydroxymethyltransferase of *Lactobacillus murinus* (32 % identity with 93 % query coverage).

**Fig. 6:**
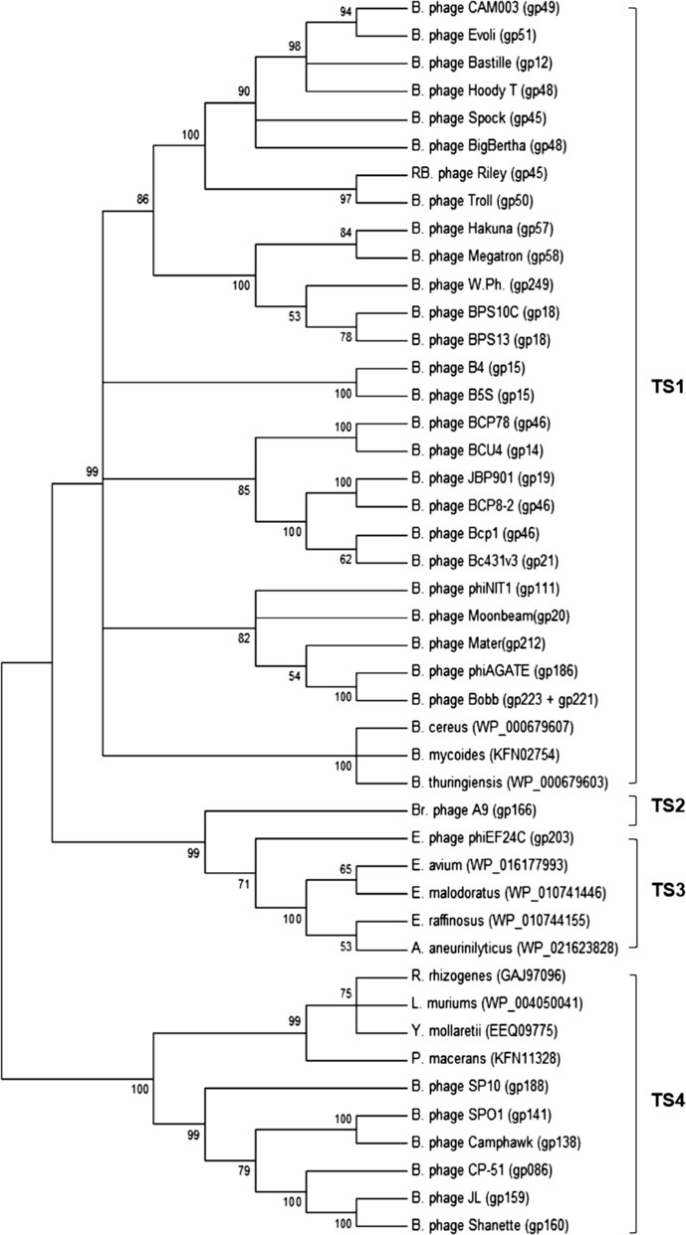
Evolutionary relationship of thymidylate synthases. The thymidylate synthase genes were compared by Muscle multiple sequence alignment, and a phylogenetic tree was generated with MEGA v6 [27] using the Maximum Likelihood (ML) method. Bootstrapping was set to 1000 and the unrooted tree was collapsed at a less than 50 % bootstrap value. A, *Aneurinibacillus*; B, *Bacillus*; Br, *Brochothrix*; E, *Enterococcus*; L, *Lactobacillus*; R, *Rhizobium*; P, *Paenibacillus*; S, *Staphylococcus*; Y, *Yersinia*

When the amino acid sequences of TS from *Spounavirinae* phages and bacteria were phylogenetically analyzed, four types of TS were clearly observable; TS1 containing TS from Bastille-like group phages and *Bacillus* spp., TS2 from *Brochothrix* phage A9, TS3 from phiEF24C and *Enterococcus* spp., and TS4 from SPO1-like group phages, SP10 and other bacteria such as *Rhizobium rhizogenes*, *Lactobacillus muriums*, *Yersinia mollaretii* and *Paenibacillus macerans* (Fig. 6).

### A putative dihydrofolate reducatase (DHFR) homolog is unique to Bastille-like group phages and serves as additional signature gene for the group

Pham 369 contains 26 gene products (Fig. 4b) which are found in, and are restricted to, all 26 Bastille-like group phages. BLASTP analysis using 26 gene products in pham 369 against 35 non-Bastille-like group phages did not return any protein with significant homology. Global identities and E-values among the 25 gene products (except phage B4; See below) are 38.9–100 % and 3.87 × 10^−37^ – 5.95 × 10^−119^, respectively. When the 25 gene products in pham 369 were BLAST-searched, significant hits of DHFR proteins from *Bacillus* spp. (*B. cereus, B. thuringiensis* and *B. mycoides*) were identified (34–41 % amino acid identity).

**Fig. 7:**
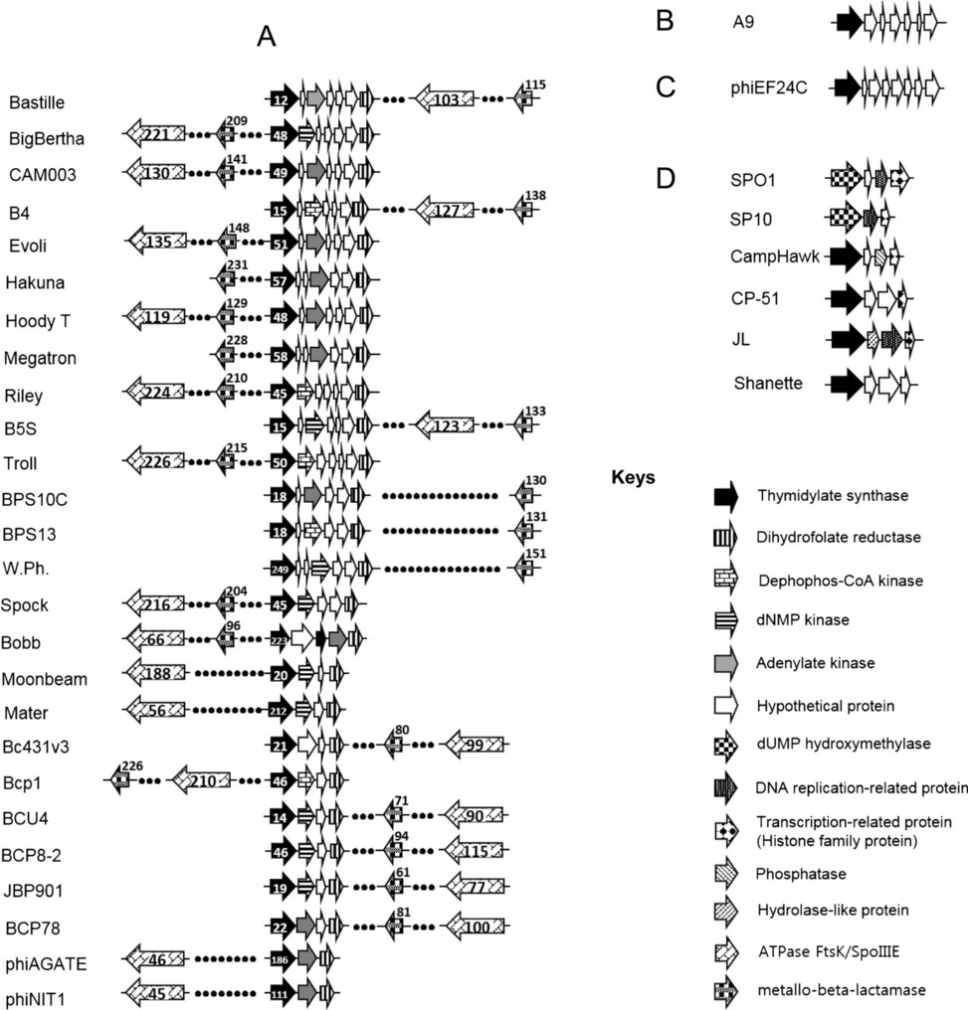
Gene arrangement of TS, DHFR, SpoIIIE and beta-lactamase genes in phage genomes. **a** Bastille-like group; **b** Singleton; **c** Twort-like group; **d** SPO1-like group. Gene location is not to the scale. Numbers found in (A) represent gene designation number in each genome

B4 *gp21* encoded a putative DHFR, however, the gene size (71 amino acids long) as annotated in the published genome was much smaller than the average size (164.9 amino acids) of DHFR proteins from other members of the Bastille-like phages. When the B4 genome was compared with B5S due to their high similarity [24], both phages share 100 % nucleotide identity in the DHFR region. We further observed that the difference in DHFR gene sizes was due to the choice of start codon (ATG and TTG for B4 and B5S, respectively) used in predicting the ORFs in both cases. Accordingly, the open reading frame of B4 DHFR was modified and used for preparing Fig. 7 The new gp21 of phage B4 shares a minimum global identity and E-value of 47.3 % and 2.71 × 10^−51^, respectively, to the DHFR in the other Bastille-like phages.

### All the members of Bastille-like group phages encode metallo-beta-lactamase and/or SpoIIIE homologs

The proteins in pham 473 were found in only 21 members of Bastille-like group phages. These genes encode a SpoIIIE homolog with a minimum identity of 40.0 % among the pham members (Fig. 4c). BLASTP analysis indicated that the SpoIIIE of the phages in the Bastille-like group shared at least 29 % identity (E-value 2 × 10^−72^ and 82 % query cover) with DNA translocase stage III sporulation protein of *B. cereus*.

Metallo-beta-lactamase gene products (pham 484) were identified in 23 members of the Bastille-like group with a minimum amino acid identity of 28.8 % (E-value of 4 × 10^−48^)*.* Bioinformatic analysis revealed that the metallo-beta-lactamase protein of Bastille-like group members shared at least 25 % identity (E-value of 1 × 10^−9^ and 93 % query coverage) with metal-dependent hydrolase of *B. thuringiensis*. Pham 484 was also found in *Bacillus* phage SP10 (gp217) (Fig. 4c) and shares between 28.8 % and 36.8 % identity with the Bastille-like group phages.

In addition to pham 484*,* pham 672 contained metallo-beta-lactamase domain that was found only in *Bacillus* phage Mater (gp126), a member of Bastille. BLAST analysis indicated that gp126 of phage Mater shares 46 % amino acid sequence identity, an E-value 1 × 10^−59^, and 95 % coverage with beta-lactamase of the facultative anaerobic, endospore-forming *Paenibacillus mucilaginosus* (formerly *Bacillus mucilaginosus*) [22]*.*

Putative SpolllE and beta-lactamase homologs are found close each other, 10 (B5S and Hoody T) to 30 ORFs (Spock) apart and are located within the nucleotide metabolism, replication and transcription modules containing putative RNA polymerase sigma factor, chromosome segregation protein, RNA ligase, and plasmid segregation protein (Fig. 7; data not shown). With respect to TS1 and DHFR region, putative SpoIIIE and beta-lactamase genes were transcribed in the opposite orientation (Fig. 7a).

## Discussion

The *Spounavirinae* are currently composed of the Spouna-like virus (SPO1-like) group (SPO1), the Twort-like virus group (G1, A511, P100, K and Twort) and unassigned phages (phiEF24C and LP65). Recently, Barylski et al. (2014) [3] proposed a third group, Bastille-like viruses, containing eight members. In addition to eight ICTV-recognized *Spounavirinae* phages, we collected the genomes of all 53 putative subfamily members currently published as described in Materials and Methods. When their genome relatedness was analyzed at nucleotide as well as protein levels, three groups were clearly observed.

The SPO1-like group contains 5 members (SPO1, CP-51, CampHawk, Shanette and JL) that infect either *B. cereus* or *B. subtilis*. The Twort-like group, includes 26 members (including phage Twort, K, G1, P100, A511, AG20, phiEF24C, Remus, Romulus, GH15, JD007 and others) with different hosts (*Staphylococcus, Listeria, Lactobacillus* and *Enterococcus*). The novel third group (Bastille-like) includes 26 *Bacillus* phages. Finally four phages, A9, SP10, Lb338-1 and LP65, remain orphan phages in the subfamily. Revisiting the data presented in other publications, which followed the current ICTV-approved classification [20], also supports the presence of a third distinct group among the *Spounavirinae*. Together with the previous publication on the possible existence of the third group [3], this convincing evidence prompts for a revision of the grouping within the *Spounavirinae* subfamily.

The Bastille-like group phages were isolated from soil, sewage or food using *B. cereus* (12 phages), *B. thuringiensis* (10 phages), *B. subtilis* (1 phage), *B. megatrium* (2) or *B. pumilus* (1 phage) as host, in different geographical locations (Table 1). They feature a head diameter of 95 ± 10 nm and tail length of 185 ± 30 nm. Their genome sizes range from approx. 127 kb to 165 kb, with G + C content between 37 % and 50 %. Among the *Bacillus* phages isolated so far, members of this novel Bastille-like group represent about 29 % with an increasing number in isolations recently [13]. However, despite their growing population, there is little data on host range, genome structure, and receptors of these phages. These data would be required for more complete classification (Table 1).

**Table 1:** Characteristics of Bastille-like group phages.

Phage name	Original host	Isolation Map	Sample of isolation	Genome size	Head diameter	Tail length	GC%	Predicted ORFs	tRNAs	Accession number	Ref.
BCP8-2	*B. cereus*	S. Korea	fermented food	159071	95	210	39.4	220	18	KJ081346	[28]
B4	*B. cereus*	S. Korea	mud sample	162596	85	213	37.7	277	0	JN790865	[22]
B5S	*B. cereus*	S. Korea	ND	162598	ND	ND	37.7	272	0	JN797796	[17]
BCP78	*B. cereus*	S. Korea	fermented food	156176	ND	ND	39.9	227	18	JN797797	[37]
BCU4	*B. cereus*	S. Korea	ND	154371	ND	ND	39.9	223	19	JN797798	[17]
BPS10C	*B. cereus*	S. Korea	ND	159590	ND	ND	38.7	271	0	NC_023501	[38]
BPS13	*B. cereus*	S. Korea	ND	158305	ND	ND	38.8	268	0	JN654439	[38]
Bastille	*B. cereus*	Canada	ND	153962	90	200	38.1	280	7	JF966203	[20]
Bcp1	*B. cereus*	USA	landfill soil	152778	ND	ND	39.8	227	17	KJ451625	[12]
JBP901	*B. cereus*	S. Korea	fermented food	159492	95 ± 5	170 ± 5	39.7	201	19	KJ676859	[24]
W.Ph.	*B. cereus*	Switzerland	ND	156897	90	203	36.5	274	0	HM144387	[20]
Bc431v3	*B. cereus*	Egypt	sewage	158621	85.4 ± 3	180 ± 3	40.0	238	21	JX094431	[10]
phiAGATE	*B. pumilus*	Poland	water samples	149844	91.16 ± 3.71	165.41 ± 8.67	50.0	210	4	NC_020081	[3]
BigBertha	*B. thuringiensis*	USA	soil sample	165238	ND	ND	37.8	291	0	NC_022769	[39]
CAM003	*B. thuringiensis*	USA	ND	160541	ND	ND	38.0	296	8	KJ489397	-
Evoli	*B. thuringiensis*	USA	ND	159656	ND	ND	38.1	293	8	KJ489398	-
Hakuna	*B. thuringiensis*	USA	ND	158100	ND	ND	38.7	294	0	NC_024213	-
HoodyT	*B. thuringiensis*	USA	ND	159837	ND	ND	38.0	299	8	KJ489400	-
Megatron	*B. thuringiensis*	USA	ND	158750	ND	ND	38.8	291	0	NC_024211	-
Riley	*B. thuringiensis*	USA	ND	162816	ND	ND	37.8	290	0	NC_024788	-
Spock	*B. thuringiensis*	USA	soil sample	164297	ND	ND	37.6	283	0	NC_022763	-
Troll	*B. thuringiensis*	USA	ND	163019	ND	ND	37.8	289	0	NC_022088	-
Moonbeam	*B. megatrium*	USA	soil sample	161239	ND	ND	40.2	231	3	KM236246	[40]
Mater	*B. megatrium*	USA	Soil sample	164302	ND	ND	39.5	222	6	KM236245	[1]
phiNIT1	*B. subtilis*	Japan	ND	155631	ND	ND	42.1	219	4	NC_021856	-

The creation of a Phamerator database using 61 published large-genome *Spounavirinae* helped to identify the Bastille-like group-specific gene products, which are TS1 and DHFR homologs. These gene products are found in all 26 members of the group but not in other groups of the subfamily. While TS1 exhibits high similarity (at least 50 %) with *Bacillus* TS, other TS types (TS2, TS3 and TS4) contain higher similarities with non-*Bacillus* TS, indicating different origins of TS1 and the other types of TS. In addition, DHFR homologs are not found in other phages analyzed in this study than the members of the Bastille-like group in the database. We propose that TS1 and DHFR can be used as signature genes that can distinguish Bastille-like group from other groups in *Spounavirinae.*

Thymidylate synthase (TS) is a folate-binding enzyme which catalyzes the transfer of one carbon unit to dUMP using 5,10-methylene-5,6,7,8-tetrahydrofolate (CH_2_H_4_folate) as a cofactor to produce dTMP and 7,8-dihydrofolate (H_2_folate) [7]. dUMP hydroxymethylase (dUMP-HMase) also catalyzes one carbon transfer from CH_2_H_4_folate to dUMP but produces hydroxymethyl-dUMP and 5,6,7,8-tetrahydrofolate (H_4_folate) [22]. Due to their functional differences, TS (but not dUMP-HMase) activity requires replenishment of H_4_ folate which is catalyzed by dihydrofolate reductase (DHFR) [7].

Previously, dUMP HMase was suggested as the signature gene for SPO1-like phages which synthesizes hydroxymethyluracil (HMU) instead of thymidine [19]. In this study we found that dUMP-HMase may not be exclusive to the SPO1-like phages as it appears to present in non-SPO1-like phage, SP10. Although SP10 is outside the SPO1-like group phages its genome appears to be modified as reported previously [36]. Therefore the presence of dUMP-HMase may still be a signature of dHMU-modified phage genomes.

A more detailed analysis indicated that TS1 proteins in Bastille-like phages are 289–311 amino acids long, which is significantly smaller than TS3 (315 amino acids long) or TS4 (382–407 amino acids long) (data not shown) but within the range of TS2 (304 amino acids long). In addition, while all TS1 genes in Bastille-like phages and in bacteria (*B. cereus*, *B. mycoides*, *B. thuringiensis*) were annotated as thymidylate synthase, those in TS4 were annotated differently in the database. While TS4 in phages such as CampHawk and JL, and *Paenibacillus macerans* has been annotated as a thymidylate synthase, in other phages (SPO1, SP10) and *Yersinia mollaretii* is was proposed as a dUMP hydroxymethylase (data not shown). Moreover, TS4 in Shanette, CP-51 and *Lactobacillus murinus* were annotated replication protein, and hypothetical protein, respectively. This finding again underlines the importance of developing universal guidelines for genome annotation. Interestingly, all the Bastille-like group phages (but no other *Spounavirinae* phages) contain a dihydrofolate reductase near the TS1. These data collectively suggest that TS4 is different from TS1 and might contain dUMP HMase activity. In addition, *Enterococcus* phage phiEF24C and *Brochothrix* phage A9 also does not encode a DHFR gene in its genome. Clearly, more studies will be required for clarification of the functional differences between TS1 and TS4 (Fig. 7).

Bacteriophage genomes usually exhibit a modular structure in which related-genes form a module, which might be transferred together from one phage to another [5, 35]. Thus, it is likely that closely related-phages have a similar gene arrangement. The TS1-DHFR region in all the Bastille-like phages exhibits a nearly identical gene arrangement (Fig. 7). The TS1-DHFR region is preceded by putative terminase and endolysin genes, approximately 10 and 12 ORFs in the same orientation, respectively. In addition, TS1 is followed by the dihydrofolate reductase gene, two to six ORFs apart, in the same orientation (Fig. 7a). On the other hand, putative recombinase (gp150) and putative DNA-binding protein (gp178) are found upstream and downstream of TS2 (gp166), respectively, in phage A9. The genes upstream of *gp203* and the TS3 region in phiEF24C include putative ribonucleotide reductases in the same orientation, while putative terminase and endolysin genes are 17 and 22 ORFs apart in an opposite orientation (data not shown). In addition, the region downstream of gp203 does not contain a DHFR encoding gene (Fig. 7b). TS4 genes are located in the middle of the DNA replication/transcription module, usually encoding such enzymes as DNA polymerase, Sigma factors, RNA polymerase, nuclease and primase (data not shown). These data suggest that TS1 in the Bastille-like group originated from the same source, which might be different from the source of TS2, TS3 and TS4 genes.

Based on results obtained from CLANS (Fig. 1), dot plot (Fig. 2), phylogenetic analysis using single gene products (Fig. 3a and b), and CoreGenes analysis of the proteome (data not shown), *Bacillus* phage Bobb is consistently classified as a member of the Bastille-like group. Interestingly, two TS-encoding genes (*gp221* and *gp223*) were detected in its genome. Further analysis suggested that *gp221* and *gp223* could be a part of a gene separated by insertion of *gp222*, an intron endonuclease homolog (Fig. 5). A similar genotype was reported by Bechhofer et al. (1994) [4] when they discovered an intron in the thymidylate synthase homolog of a broad host range *Bacillus* phage beta 22. The origin of these intron endonuclease homologs in both bacteria and phages has not been fully elucidated. Nevertheless, either gp223 or a combined protein of gp223 and gp221 was clustered together with other Bastille-like group phages in phylogenetic tree, validating TS1 as a signature gene in the group.

Bastille-like and SPO1-like group phages share the same hosts, *Bacillus* spp. Thus, the clear distinction of the two groups based on TS1-DHFR region is very intriguing. Other phylogenetic studies such as nucleotide sequence-based CLANS and single protein sequence-based phylogenetic trees separate Bastille-like and SPO1-like groups. Therefore, it might be suggested that the acquisition of the two signature genes is as old as the divergence of the two *Spounavirinae* groups. In addition, it could provide a possible explanation for the diversities in the Bastille-like group phages which was further developed by vertical gene transfer followed by mutations in the region.

Although all the Bastille-like phages encode TS1 and DHFR with significant identities to the TS1 and DHFR of *Bacillus cereus*, the gene arrangement inside the TS1-DHFR region is different. No phage contained TS1 and DHFR genes right next to each other, as observed in most bacteria including *Bacillus* spp. (data not shown). There are one (phiAGATE and phiNIT1) to five putative ORFs (Bastille and others) in between the genes (Fig. 7). Taken together, these data suggest that not only vertical gene transfer but also horizontal gene transfer might take place in this region [15, 16].

TS and DHFR are found in the genome of *Bacillus* spp. and not all the *Bacillus* phages have these genes. On the other hand, TS and DHFR are commonly found in all Bastille-like group phages and appear to be a part of the phage genome for a long time. This could imply that these genes somehow play an important role in lifecycle or physiology of Bastille-like phages. Currently, however, the function(s) and the meaning of the products encoded by these two genes in phage genome are unknown and are certainly of great interest for further studies.

Due to relatively broad host range and strictly virulent phenotype, *Spounavirinae* viruses received an increasing interest as tools to control harmful bacteria [6]. It is generally accepted that phages intended for biocontrol should not feature genes which encode putative virulence factors or may possibly enhance the pathogenic profile of the target bacteria [14]. Phage genome analysis has allowed for convenient detection of phages encoding or lacking putative virulence factors. In this study, we found that all the members of Bastille-like group phages encode a metallo-beta-lactamase protein and/or a SpoIIIE homolog, which might play a role in host virulence or pathogenesis.

Recently, Colomer-Lluch et al (2011) reported that phage-encoded antibiotic resistance genes can confer resistance to bacteria depending on the strain and environmental factors [8]. In addition, a number of phages, including *Bacillus* phage SP10, were reported to affect sporulation efficiency of the host [31]. Putative sporulation-related genes in some of the members of Bastille-like phages were reported previously [10, 23, 30]. However, no experiments have proven that phage-encoded gene products are directly related to bacterial sporulation. More studies are required to clarify the role of these genes in the phage genomes. Despite the lack of clear evidence that identifies the role of these phage gene products in host virulence, their presence makes the use of these phages questionable as biocontrol agents.

Previously, the genomes of two members of the proposed SPO1-like group phages, JL and Shanette, were also reported to encode tellurium resistance protein [12]. Interestingly, none of the Twort-like phages have so far been reported to contain genes encoding for antibiotic resistance or host survival. These data suggest that, while the functional identity of these genes needs to be verified experimentally for the future, Twort-like phages might be better candidates as biocontrol agents.

## Conclusions

In summary, we analyzed 61 complete genome sequences of *Spounavirinae* phages and confirmed the creation of the “Bastille-like group” in the subfamily. Furthermore TS1- and DHFR-encoding genes were identified to be unique in *Spounavirinae*, which could serve as signatures for the new Bastille-like group.

## Methods

### Data collection

Sixty-one prospective *Spounavirinae* phages were recruited and included in the analysis (33 NCBI taxonomy-classified [ID*:* 857473], 27 *Bacillus* Myoviridae phages with a genome size greater than 127 kb, and one *Lactobacillus* phage Lb338-1).

As of the date of manuscript submission, the NCBI taxonomy-classified *Spounavirinae* includes 33 members whose full genomic information is available. This contains eight ICTV classified *Spounavirinae* phages (*Bacillus* phage SPO1 [GenBank Accession Number NC_011421], *Staphylococcus* phages Twort [NC_007021], G1 [NC_007066] and K [KF766114], *Listeria* phages A511 [NC_009811] and P100 [DQ004855], *Enterococcus* phage phiEF24C [AP009390] and *Lactobacillus* phage LP65 [NC_006565]). It also includes 25 ICTV-unclassified phages (four *Bacillus* phages Bastille [NC_018856], BCP8-2 [KJ081346.1], CP-51 [NC_025423], Bc431v3 [NC_020873]; 19 *Staphylococcus* phage Sb-1 [NC023009], 676Z [JX080302], A35 [JX080301], A5W [EU418428], Fi200W [JX080303], ISP [FR852584], MSA6 [JX080304], P4W [JX080305], SA5 [JX875065], Staph1N [JX080300], GH15 [NC019448], JD007 [NC_019726], MCE-2014 [NC_025416], P108 [NC_025426], phiSA012 [NC_023573], S25-3 [NC_022920], S25-4 [NC_022918], Remus [NC_022090], and Romulus [NC_020877]; one *Listeria* phage AG20 [NC_020871]; one *Brochothrix* phage A9 [NC_015253]).

In addition, 27 complete genome sequences of candidate *Spounavirinae Bacillus* phages were collected from NCBI database. This includes B5S [JN797796], Spock [NC_022763], B4 [JN790865], Riley [NC_024788], Troll [NC_022088], BigBertha [NC_022769], Hoody T [NC_024205], Evoli [NC_024207], CAM003 [NC_024216], W.Ph. [NC_016453], BPS13 [NC_018857], BPS10C [NC_023501], Megatron [NC_024211], Hakuna [NC_024213], JBP901 [KJ676859.1], Bcp1 [NC_024137], BCP78 [NC_018860], BCU4 [JN797798], phiNIT1 [NC_021856], Mater [KM236245], Moonbeam [KM236246], phiAGATE [NC_020081], Bobb [NC_024792], SP10 [NC_019487], CampHawk [NC_022761], Shanette [KC595513], and JL [KC595512].

As of the date of manuscript submission, there are three more complete genome sequences of *Bacillus* phages (Grass [NC_022771], G [NC_023719] and 0305phi8-36 [NC_009760]) available in NCBI database whose genome is bigger than 127 kb. However, they were excluded from the analysis because of either the lack of information (the family of phage Grass is not specified) or significantly bigger genome size (approx. 219 and 498 kb for 0305phi8-36 and G, respectively).

Furthermore, *Lactobacillus* phage Lb338-1 was included in all the analysis since it has been reported as an SPO1-like phage [1, 19].

### Phylogenetic analysis

The **CL**uster **An**alysis of **S**equences (CLANS) software package [11] was used to compare all 61 members using BLASTn as described previously [3]. It uses the Fruchterman and Reingold graph layout algorithm to generate graphs after performing all-against-all BLAST searches, and calculating pairwise attraction values based on the *P*-values of high scoring segment pairs (HSPs) [11]. Dot plots of protein sequences were generated using Gepard [21]. We also constructed maximum likelihood (ML) trees using protein sequences from the 61 phages (putative major capsid protein, tail sheath protein, large terminase subunit and DNA polymerase) as described previously [10]. Bootstrapping was set to 1000 and the unrooted tree was collapsed at a less than 50 % bootstrap value. The tree was drawn using Mega v6.0 [32].

### Phamerator database analysis

The Phamerator database was created as described previously [9]. It uses BLASTP [2] and ClustalW [33] to compare each putative protein from all phages in the user-created database [27]. The percent identities and BLASTP E-value scores are used to sort proteins into phamilies (phams) based on user-defined cutoffs for each score [9]. Conserved domains in each protein are then identified. The database used in this study consists of 61 large genome phages as described above. Proteins were grouped into phamilies (phams) when they exhibited a BLASTP E-value lower than 1.0 x 10^−50^ or greater than 32.5 % identity with at least one other protein [9, 27]. Conserved domains in each protein were identified using RPS-BLAST [26]. Pham circles were drawn with Phamerator program.

### Availability of supporting data

The dataset supporting the results of this article is included within the additional files (Additional file 1, the complete dot plot analysis; Additional file 2, Phamerator database containing the 61 phage genomes; Additional files 3 and 4, the spreadsheet exported from the Phamerator database to show all phage gene products, the phams, and the conserved domains found in those phams).

## Additional files

Additional file 1: Figure S1. Nucleotide (A) and amino acid sequence (B) dot plot analysis of 61 *Spounavirinae* phage genomes. Additional file 2:
**The Phamerator database for this study (61phages).**
Additional file 3:
**Cluster table of phamerator analysis.**
Additional file 4:
**Pham table of phamerator analysis.**

## Abbreviations

BLAST: Basic local alignment
CLANS: Cluster analysis of sequences
CRISPR: Clustered regularly interspersed short palindromic repeats
DHFR: Dihydrofolate reductase
ICTV: International committee on taxonomy of viruses
dUMP-HMase: Deoxyuridine monophosphate hydroxymethylase
HMU: Hydroxymethyluracil
NCBI: National center for biotechnology information
TS: Thymidylate synthase
