# Supplementary material for: Putative type 1 thymidylate synthase and dihydrofolate reductase as signature genes of a novel bastille-like group of phages in the subfamily Spounavirinae
Source: BMC Genomics. 2015 Aug 7;16(1):582. doi: 10.1186/s12864-015-1757-0 (PMC4528723; doi:10.1186/s12864-015-1757-0)
Supplement: Additional file 1: Figure S1. — Nucleotide (A) and amino acid sequence (B) dot plot analysis of 61 Spounavirinae phage genomes. [file 12864_2015_1757_MOESM1_ESM.pptx]

## Slide 1
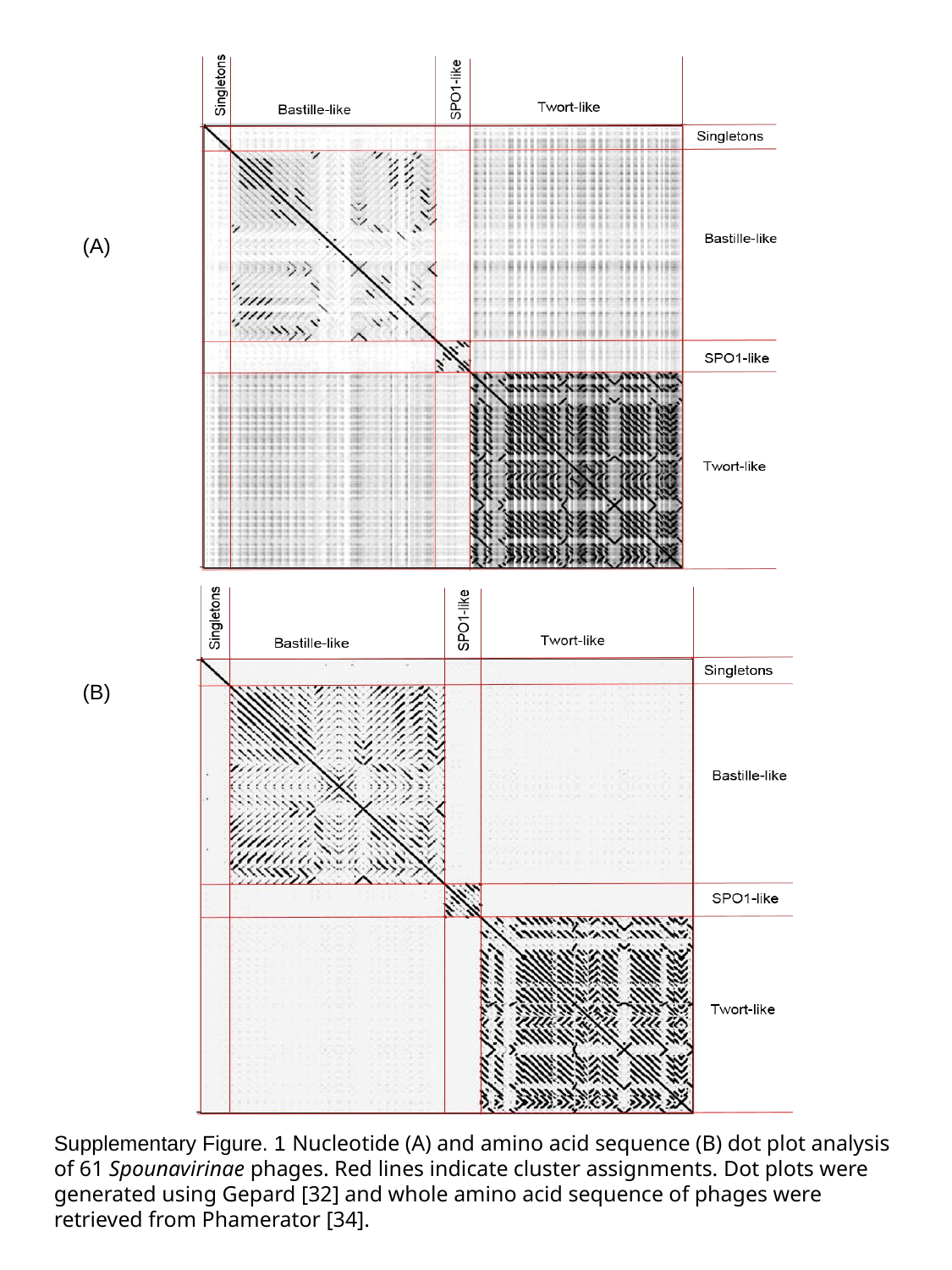

(A)
(B)
Supplementary Figure. 1 Nucleotide (A) and amino acid sequence (B) dot plot analysis of 61 Spounavirinae phages. Red lines indicate cluster assignments. Dot plots were generated using Gepard [32] and whole amino acid sequence of phages were retrieved from Phamerator [34].
